# Supplementary material for: Species Identification and Fungicide Sensitivity of Fusarium spp. Causing Peanut Root Rot in Henan, China
Source: J Fungi (Basel). 2025 Jun 6;11(6):433. doi: 10.3390/jof11060433 (PMC12194549; doi:10.3390/jof11060433)
Supplement: Supplementary file 1 [file jof-11-00433-s001.zip › jof-3538609-supplementary.pdf]

Table S1. Isolates from peanut root rot included in this study.

Species	Isolate	Host	Country	GenBank accession number				
				ITS	*TEF1-a*	*RPB2*	*TUB2*	*CAM*
Fusarium armeniacum	RZMDPY-14	*Arachis hypogaea*	Zhumadian, Henan, China	PV597952	PV607877	PV631026	PV631070	PV631103
Fusarium armeniacum	RZMDPY-18	*Arachis hypogaea*	Zhumadian, Henan, China	PV597953	PV607878	PV631027	PV631071	PV631104
Fusarium armeniacum	RZMDSC-28	*Arachis hypogaea*	Zhumadian, Henan, China	PV597954	PV607879	PV631028	PV631072	PV631105
Fusarium pseudograminearum	RPYQF-25	*Arachis hypogaea*	Puyang, Henan, China	PV596285	PV607846	PV607900	PV631041	PV631123
Fusarium pseudograminearum	RHBXX-37	*Arachis hypogaea*	Hebi, Henan, China	PV596286	PV607847	PV607901	PV631042	PV631124
Fusarium pseudograminearum	RPYQF-26	*Arachis hypogaea*	Puyang, Henan, China	PV596287	PV607848	PV607902	PV631043	PV631125
Fusarium pseudograminearum	RPYWDS-3	*Arachis hypogaea*	Puyang, Henan, China	PV596288	PV607849	PV607903	PV631044	PV631126
Fusarium graminearum	RZMDQS-11	*Arachis hypogaea*	Zhumadian, Henan, China	PV597900	PV607867	PV631016	PV631073	PV631111
Fusarium graminearum	RZMDSC-16	*Arachis hypogaea*	Zhumadian, Henan, China	PV597901	PV607868	PV631017	PV631074	PV631112
Fusarium graminearum	RPYQF-61	*Arachis hypogaea*	Puyang, Henan, China	PV597902	PV607869	PV631018	PV631075	PV631113
Fusarium graminearum	RZMDQS-24	*Arachis hypogaea*	Zhumadian, Henan, China	PV597903	PV607870	PV631019	PV631076	PV631114
Fusarium graminearum	RZMDSC-29	*Arachis hypogaea*	Zhumadian, Henan, China	PV597904	PV607871	PV631020	PV631077	PV631115
Fusarium acuminatum	RXYPQ-35	*Arachis hypogaea*	Xinyang, Henan, China	PV597919	PV607880	PV631011	PV631060	PV631098
Fusarium acuminatum	RHBXX-32	*Arachis hypogaea*	Hebi, Henan, China	PV597920	PV607881	PV631012	PV631061	PV631099
Fusarium acuminatum	RNYXY-16	*Arachis hypogaea*	Nanyang, Henan, China	PV597921	PV607882	PV631013	PV631062	PV631100
Fusarium acuminatum	RPYQF-15	*Arachis hypogaea*	Puyang, Henan, China	PV597922	PV607883	PV631014	PV631063	PV631101
Fusarium acuminatum	RNYFC-28	*Arachis hypogaea*	Nanyang, Henan, China	PV597923	PV607884	PV631015	PV631064	PV631102
Fusarium commune	RZKFG-2-3	*Arachis hypogaea*	Zhoukou, Henan, China	PV597910	PV607872	PV631021	PV631065	PV631106
Fusarium commune	RXYHB-2	*Arachis hypogaea*	Xinyang, Henan, China	PV597911	PV607873	PV631022	PV631066	PV631107
Fusarium commune	RNYNZ-2-13	*Arachis hypogaea*	Nanyang, Henan, China	PV597912	PV607874	PV631023	PV631067	PV631108
Fusarium commune	RNYWC-2-18	*Arachis hypogaea*	Nanyang, Henan, China	PV597913	PV607875	PV631024	PV631068	PV631109
Fusarium commune	RZMDZY-2-2	*Arachis hypogaea*	Zhumadian, Henan, China	PV597914	PV607876	PV631025	PV631069	PV631110
Fusarium ipomoeae	RZMDSC-17	*Arachis hypogaea*	Zhumadian, Henan, China	PV598152	PV607863	PV631032	PV631127	PV631116
Fusarium ipomoeae	RZMDSC-3	*Arachis hypogaea*	Zhumadian, Henan, China	PV598153	PV607864	PV631033	PV631128	PV631117
Fusarium ipomoeae	RZMDSC-32	*Arachis hypogaea*	Zhumadian, Henan, China	PV598154	PV607865	PV631034	PV631129	PV631118

Fusarium ipomoeae	RZMDSC-22	*Arachis hypogaea*	Zhumadian, Henan, China	PV598155	PV607866	PV631035	PV631130	PV631119
Fusarium lacertarum	RPYQF-11	*Arachis hypogaea*	Puyang, Henan, China	PV597955	PV607860	PV631029	PV631131	PV631120
Fusarium lacertarum	RPYQF-28	*Arachis hypogaea*	Puyang, Henan, China	PV597956	PV607861	PV631030	PV631132	PV631121
Fusarium lacertarum	RJZMZ-2-12	*Arachis hypogaea*	Jiaozuo, Henan, China	PV597957	PV607862	PV631031	PV631133	PV631122
Fusarium neocosmosporiellum	RXYHB-2-1	*Arachis hypogaea*	Xinyang, Henan, China	PV596645	PV607855	PV607904	PV631055	PV631093
Fusarium neocosmosporiellum	RZKXC-2-4	*Arachis hypogaea*	Zhoukou, Henan, China	PV596646	PV607856	PV607905	PV631056	PV631094
Fusarium neocosmosporiellum	RZMDSP-2-2	*Arachis hypogaea*	Zhumadian, Henan, China	PV596647	PV607857	PV607906	PV631057	PV631095
Fusarium neocosmosporiellum	RXXYY-18	*Arachis hypogaea*	Xinxiang, Henan, China	PV596648	PV607858	PV607907	PV631058	PV631096
Fusarium neocosmosporiellum	RLYYS-2-11	*Arachis hypogaea*	Luoyang, Henan, China	PV596649	PV607859	PV607908	PV631059	PV631097
Fusarium solani	RPYNL-2-7	*Arachis hypogaea*	Puyang, Henan, China	PV589479	PV598169	PV607885	PV631036	PV631078
Fusarium solani	RNYSQ-2-2	*Arachis hypogaea*	Nanyang, Henan, China	PV589478	PV598170	PV607886	PV631037	PV631079
Fusarium solani	RXXYY-2-13	*Arachis hypogaea*	Xinxiang, Henan, China	PV589481	PV598171	PV607887	PV631038	PV631080
Fusarium solani	RSQNL-2-11	*Arachis hypogaea*	Shangqiu, Henan, China	PV589480	PV598172	PV607888	PV631039	PV631081
Fusarium solani	RLYMJ-2-14	*Arachis hypogaea*	Luoyang, Henan, China	PV589477	PV598173	PV607889	PV631040	PV631082
Fusarium oxysporum	RHBXX-24	*Arachis hypogaea*	Hebi, Henan, China	PV596545	PV607850	PV607890	PV631050	PV631088
Fusarium oxysporum	RZMDSC-2-2	*Arachis hypogaea*	Zhumadian, Henan, China	PV596546	PV607851	PV607891	PV631051	PV631089
Fusarium oxysporum	RSQMQ-2-7	*Arachis hypogaea*	Shangqiu, Henan, China	PV596547	PV607852	PV607892	PV631052	PV631090
Fusarium oxysporum	RNYSQ-2-5	*Arachis hypogaea*	Nanyang, Henan, China	PV596548	PV607853	PV607893	PV631053	PV631091
Fusarium oxysporum	RLYYS-2-4	*Arachis hypogaea*	Luoyang, Henan, China	PV596549	PV607854	PV607894	PV631054	PV631092
Fusarium proliferatum	RNYDZ-2-14	*Arachis hypogaea*	Nanyang, Henan, China	PV596504	PV607841	PV607895	PV631045	PV631083
Fusarium proliferatum	RHBQX-2-6	*Arachis hypogaea*	Hebi, Henan, China	PV596505	PV607842	PV607896	PV631046	PV631084
Fusarium proliferatum	RLHLY-2-6	*Arachis hypogaea*	Luohe, Henan, China	PV596506	PV607843	PV607897	PV631047	PV631085
Fusarium proliferatum	RSQSX-2-11	*Arachis hypogaea*	Shangqiu, Henan, China	PV596502	PV607844	PV607898	PV631048	PV631086
Fusarium proliferatum	RXYPQ-21	*Arachis hypogaea*	Xinyang, Henan, China	PV596503	PV607845	PV607899	PV631049	PV631087

Table S2. Origin, culture and sequence GenBank accession numbers of strains used for phylogenetic analyses.

Species	Strain	Host	Country	GenBank accession number				
				ITS	*TEF1-a*	*RPB2*	*TUB2*	*CAM*
Fusarium pseudograminearum	CBS 131261	*Triticum aestivum*	Iran	JX162363	JX118971	JX162517	/	/
Fusarium pseudograminearum	BBA 71458=DSM 116903	*Hordeum vulgare*	Australia	/	PQ260909	PQ274193	PQ274040	PQ240275
Fusarium pseudograminearum	NRRL 28062^{T}=CBS 109956=FRC R-5291=MAFF 237835	*Hordeum vulgare*	Australia	/	PQ260911	PQ274195	PQ274042	PQ240277
Fusarium neocosmosporiellum	CBS 446.93=IMI 316967=NHL 2919	Forest soil	Japan	LR583791	LR583670	LR583898	/	MW834175
Fusarium neocosmosporiellum	CBS 562.70^{T}=ATCC 32363=IMI 251387	*Arachis hypogaea* nut	Guinea-Bissau	KM231805	KM231933	KM232372	KM232067	KM231377
Fusarium neocosmosporiellum	CBS 533.65=IMI 302625	/	India	LR583792	LR583671	LR583899	/	MW834176
Fusarium solani	CBS 140079^{T}=NRRL 66304=G.J.S. 09-1466=FRC S-2364	*Solanum tuberosum*	Slovenia	KT313633	KT313611	KT313623	/	MW218088
Fusarium solani	LC3717	Soil	China	MW016736	MW620197	MW474722	MW534074	/
Fusarium solani	LC13849=LGS054	Soil	China	MW016735	MW620196	MW474721	MW534073	/
Fusarium lacertarum	LC7931	*Capsicum* sp.	China	MK280801	MK289638	MK289792	/	MK289691
Fusarium lacertarum	LC7927	*Capsicum* sp.	China	MK280838	MK289637	MK289791	/	MK289690
Fusarium lacertarum	LC7942	*Capsicum* sp.	China	MK280834	MK289643	MK289797	/	MK289696
Fusarium ipomoeae	CGMCC 3.19496T=LC12165	*Ipomoea aquatica*	China	MK280832	MK289599	MK289752	MW533878	MK289704
Fusarium ipomoeae	LC7940	*Capsicum* sp.	China	MK280798	MK289642	MK289796	MW533885	MK289695
Fusarium ipomoeae	LC13707=LGS036	Soil	China	MW016534	MW594377	MW474520	MW533879	MW574199

Fusarium commune	CBS 110090^T=AAS 156=BBA 71639=NRRL 31076	humus and P*isum sativum*	Denmark	/	AF362263	MW934368	/	/
Fusarium commune	LC13824=GXQZPSRDE4	*Musa nana*	China	MW016701	MW620162	MW474687	/	/
Fusarium commune	LC11660=HM259-R03	*Oryza* sp.	China	MW016699	MW620160	MW474685	MW534045	/
Fusarium oxysporum	CBS 221.49=IHEM 4508=LCP 39.531=NRRL 22546	*Camellia sinensis*	South East Asia	/	MH484963	MH484872	MH485054	MH484690
Fusarium oxysporum	CBS 140424	/	/	KT794176	KT794174	/	KT794173	/
Fusarium oxysporum	CBS 144134^T=P3	*Solanum tuberosum*	Germany	/	MH485044	MH484953	MH485135	MH484771
Fusarium graminearum	CBS 123657=CBS 123687=NRRL 31084	*Zea mays* corn	USA	DQ459823	PQ260861	MW233447	PQ273983	PQ240205
Fusarium graminearum	CBS 136009^T=TMW 4.0157	*Hordeum vulgare*	Germany	/	MW928838	MW928826	PQ273993	PQ240215
Fusarium graminearum	LC13775=F056	*Zea mays*	USA	MW016611	MW620072	MW474597	MW533966	/
Fusarium armeniacum	LC2809	Unidentified grass	China	MW016609	MW620070	MW474595	MW533964	/
Fusarium armeniacum	LC2797	Unidentified grass	China	MW016608	MW620069	MW474594	MW533963	/
Fusarium armeniacum	CBS 485.94^T=ATCC 90020=DAR 67507=F6963=FRC R-09335=FRC R-09372=IMI 352099=MRC 6230=NRRL 25141=NRRL 26847=NRRL 26908=NRRL 29133	Rain-damaged *Triticum aestivum* grain	Australia	AB587001	PQ260811	PQ274098	PQ273924	PQ240137
Fusarium acuminatum	JW 289003	Garden soil	Netherlands	MZ890555	MZ921908	MZ921777	/	MZ921595
Fusarium acuminatum	LC13796=GM80	*Hylotelephium erythrostictum*	China	MW016649	MW620110	MW474635	MW533995	/
Fusarium acuminatum	LC13798=LF1636	*Brassica* sp.	China	MW016651	MW620112	MW474637	MW533997	/
Fusarium proliferatum	NRRL 25089	*Rhopalosiphum padi*	USA	/	JF740718	JF741048	/	/

Fusarium proliferatum	CBS 217.76=BBA 11341=BBA 63624=DAOM 225133=IMI 202873=IMI 375339=NRRL 22944	*Cattleya pseudobulb*, hybrid	Germany	U34558	AF160280	JX171617	U34416	AF158333
Fusarium proliferatum	CBS 130179=NRRL 43617=UTHSC 03-60	Human blood	USA	MH865741	MW402023	MW402739	MW402223	MW402400
Fusarium ventricosum	CBS 748.79^{T}=BBA 62452=NRRL 20846=NRRL 22113	Soil	Germany	HQ897816	KM231924	JX171597	KM232054	KM231361

\: no sequence available in GenBank.

^{T} = Ex-type culture.

Table S3. Concentrations of the seven fungicides (prochloraz, tetramycin, tebuconazole, prothioconazole, difenoconazole, pyraclostrobin and pydiflumetofen) used in the current study.

Fungicides	Concentration (mg/L)			
	F. solani	***F. oxysporum***	***F. neocosmosporiellum***	***F. proliferatum***
Prochloraz	1, 0.5, 0.1, 0.05, 0.01, 0.005, 0.001	0.5, 0.2, 0.1, 0.05, 0.02, 0.01, 0.005	0.5, 0.2, 0.1, 0.05, 0.01, 0.005, 0.001	0.5, 0.1, 0.05, 0.01, 0.005, 0.002, 0.001
Tetramycin	1, 0.5, 0.2, 0.1, 0.05, 0.02, 0.01	5, 2, 1, 0.5, 0.2, 0.1, 0.05	1, 0.5, 0.2, 0.1, 0.05, 0.02, 0.01	5, 2, 1, 0.5, 0.2, 0.1, 0.01
Tebuconazole	10, 5, 1, 0.5, 0.1, 0.05, 0.01	5, 1, 0.5, 0.2, 0.1, 0.05, 0.01	10, 5, 1, 0.5, 0.1, 0.05, 0.01	5, 1, 0.5, 0.2, 0.1, 0.05, 0.01
Prothioconazole	10, 5, 2, 1, 0.5, 0.2, 0.1	20, 10, 5, 2, 1, 0.5, 0.2	10, 5, 2, 1, 0.5, 0.1, 0.05	20, 10, 5, 2, 1, 0.5, 0.1
Difenoconazole	20, 10, 5, 1, 0.5, 0.2, 0.1	20, 10, 5, 2, 1, 0.5 ,0.2	20, 10, 5, 1, 0.5, 0.1, 0.05	10, 5, 1, 0.5, 0.1, 0.05, 0.01
Pyraclostrobin	500, 100, 10, 1, 0.5, 0.1	500, 100, 10, 1, 0.5, 0.1	500, 100, 10, 1, 0.5, 0.1	500, 100, 10, 1, 0.5, 0.1
Pydiflumetofen	10, 1, 0.5, 0.2, 0.1, 0.01	10, 1, 0.5, 0.2, 0.1, 0.01	10, 1, 0.5, 0.2, 0.1, 0.01	10, 1, 0.5, 0.2, 0.1, 0.01

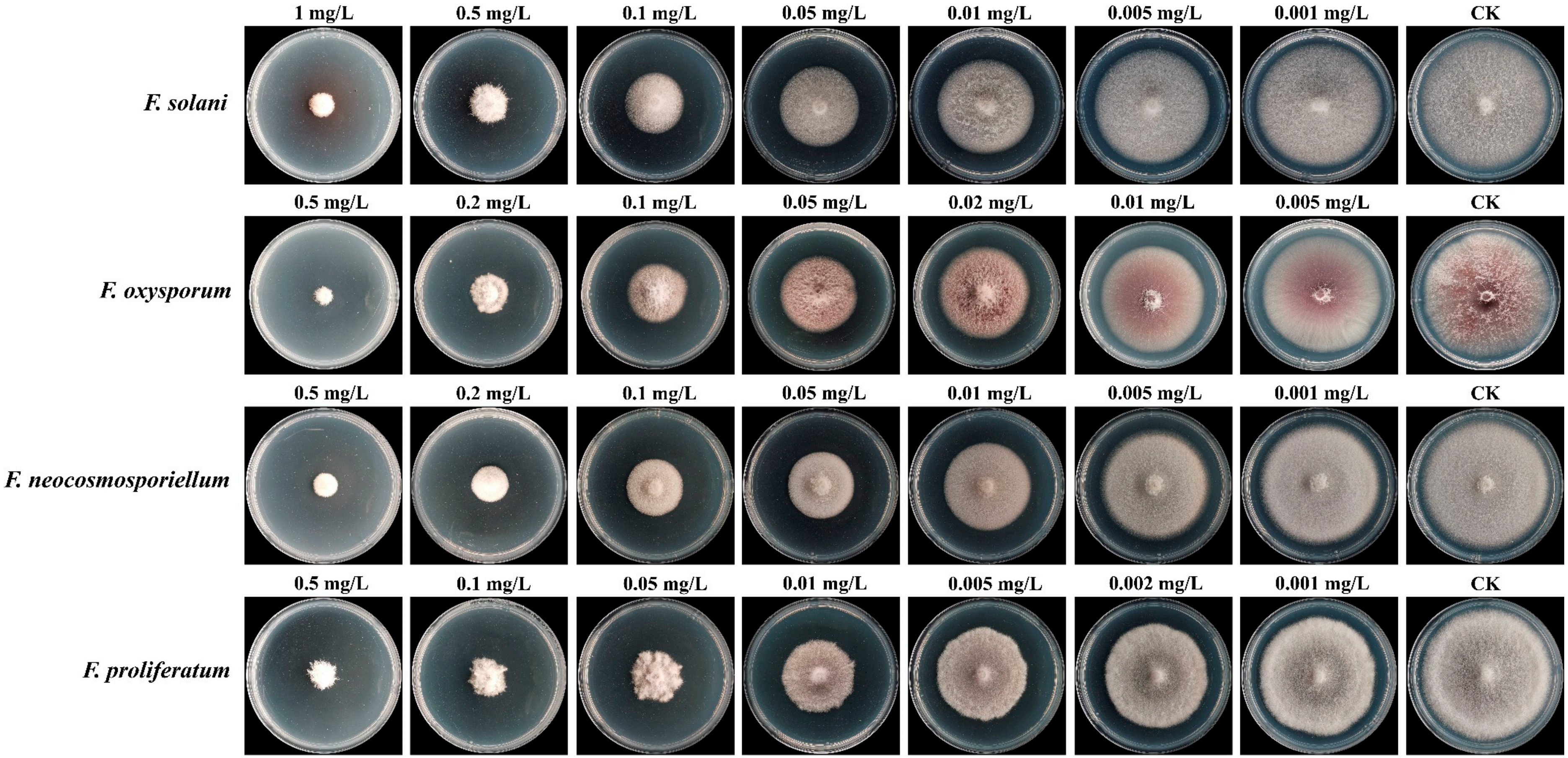

Figure S1. Effects of prochloraz on the mycelial growth of the four *Fusarium* species (*F. solani*, *F. oxysporum*, *F. neocosmosporiellum,* and *F. proliferatum*).

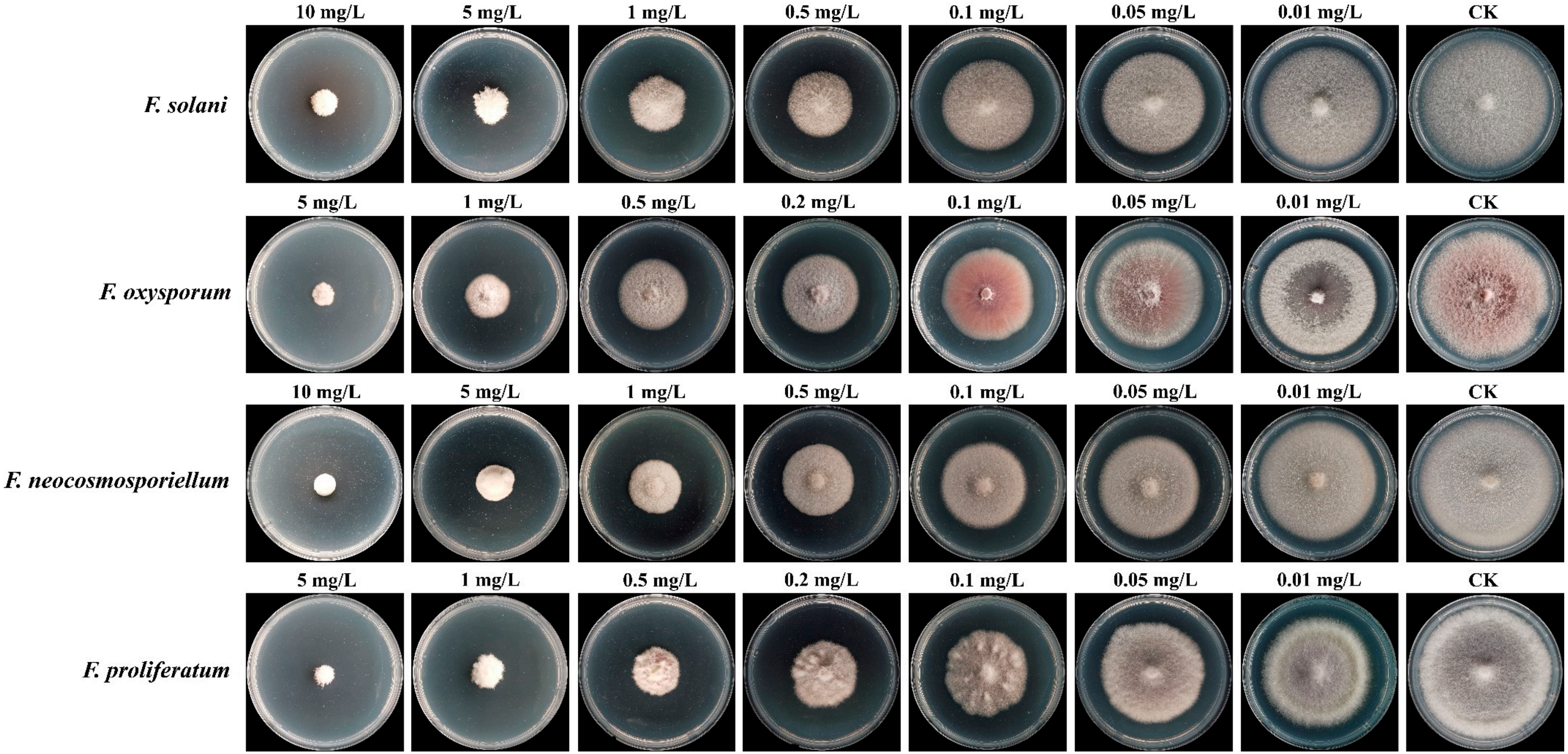

Figure S2. Effects of tebuconazole on the mycelial growth of the four *Fusarium* species (*F. solani*, *F. oxysporum*, *F. neocosmosporiellum,* and *F. proliferatum*)..

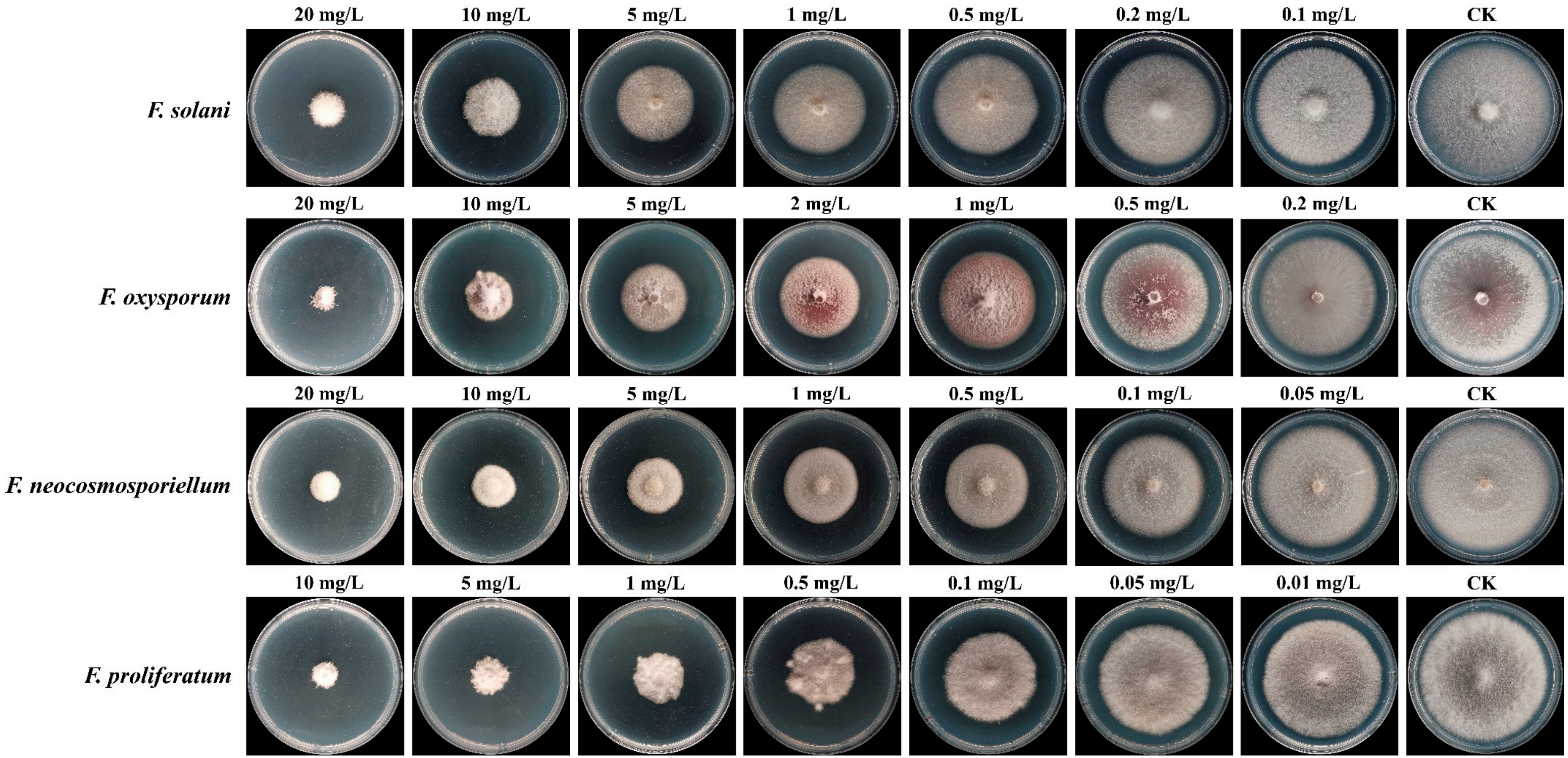

Figure S3. Effects of difenoconazole on the mycelial growth of the four *Fusarium* species (*F. solani*, *F. oxysporum*, *F. neocosmosporiellum,* and *F. proliferatum*).

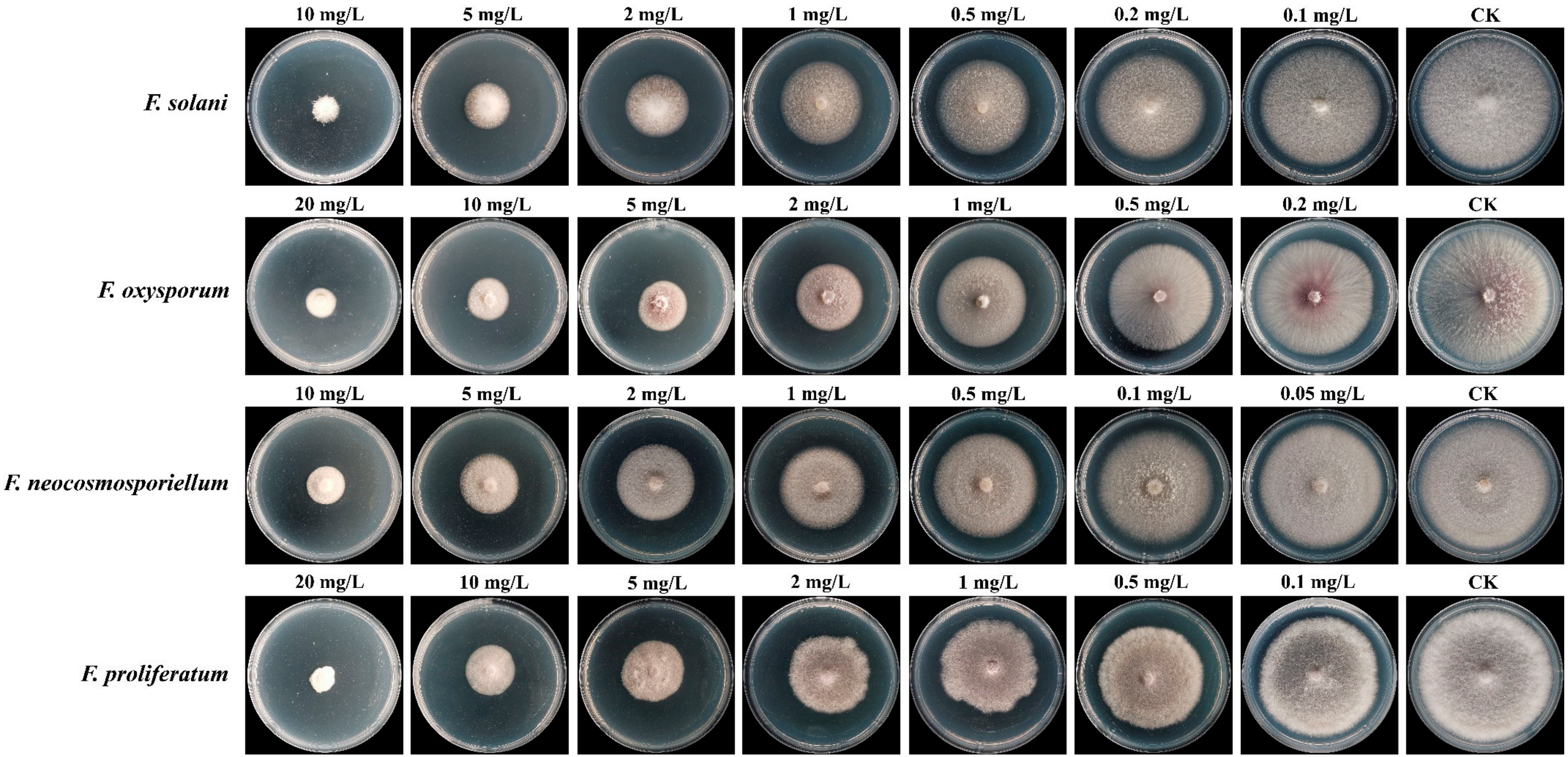

Figure S4. Effects of prothioconazole on the mycelial growth of the four *Fusarium* species (*F. solani*, *F. oxysporum*, *F. neocosmosporiellum,* and *F. proliferatum*).

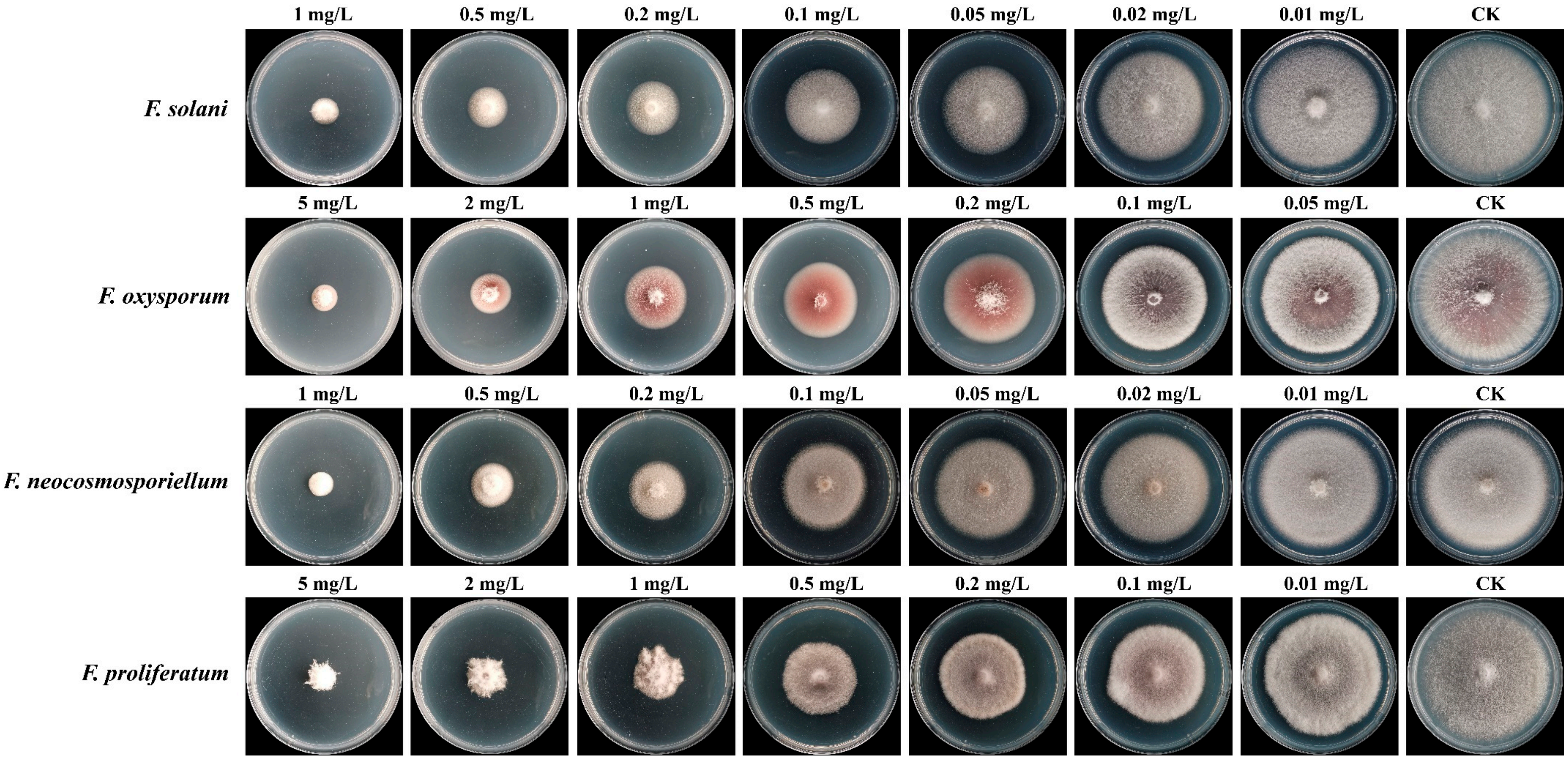

Figure S5. Effects of tetramycin on the mycelial growth of the four *Fusarium* species (*F. solani*, *F. oxysporum*, *F. neocosmosporiellum,* and *F. proliferatum*).

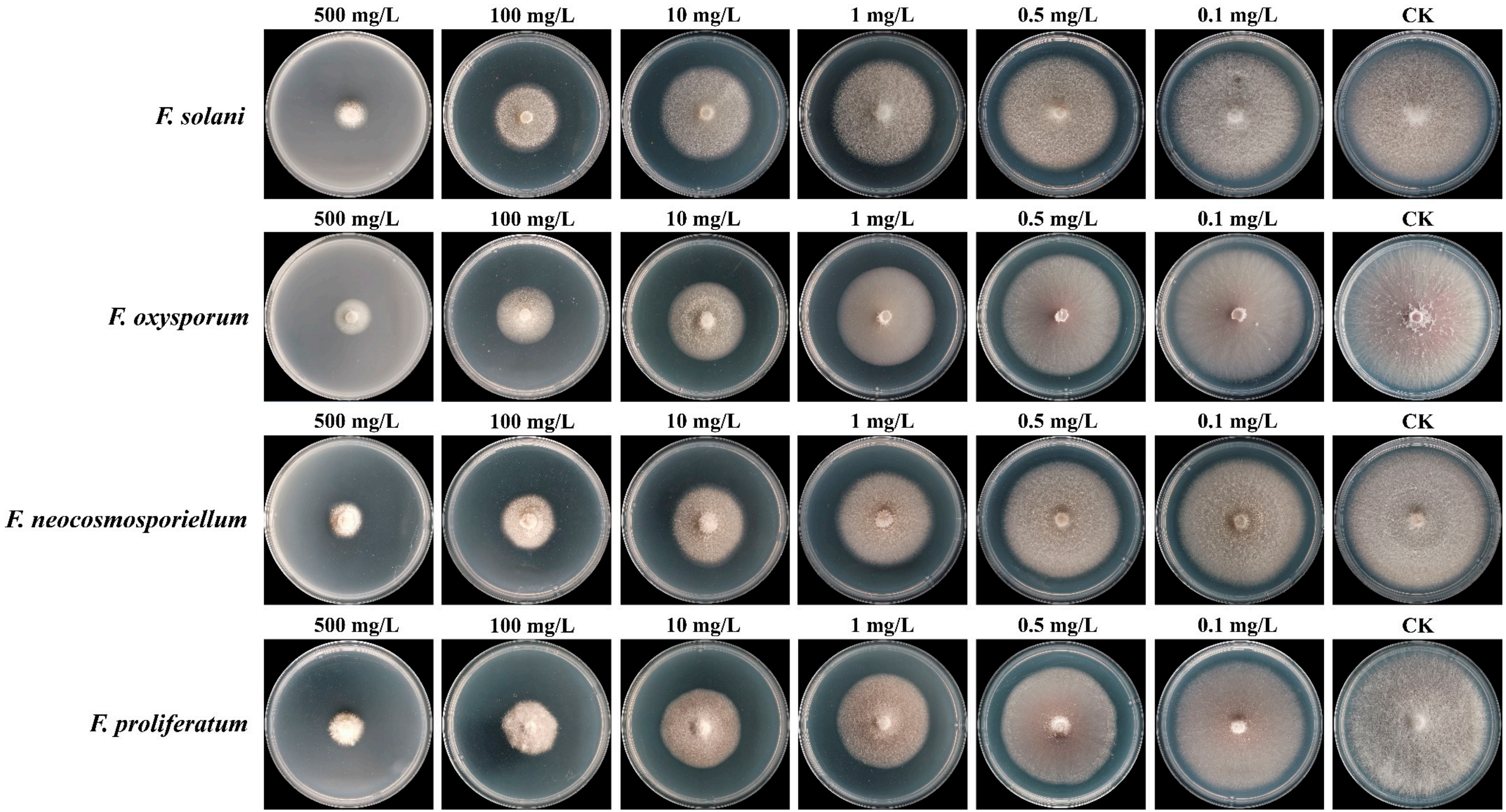

Figure S6. Effects of pyraclostrobin on the mycelial growth of the four *Fusarium* species (*F. solani*, *F. oxysporum*, *F. neocosmosporiellum,* and *F. proliferatum*).

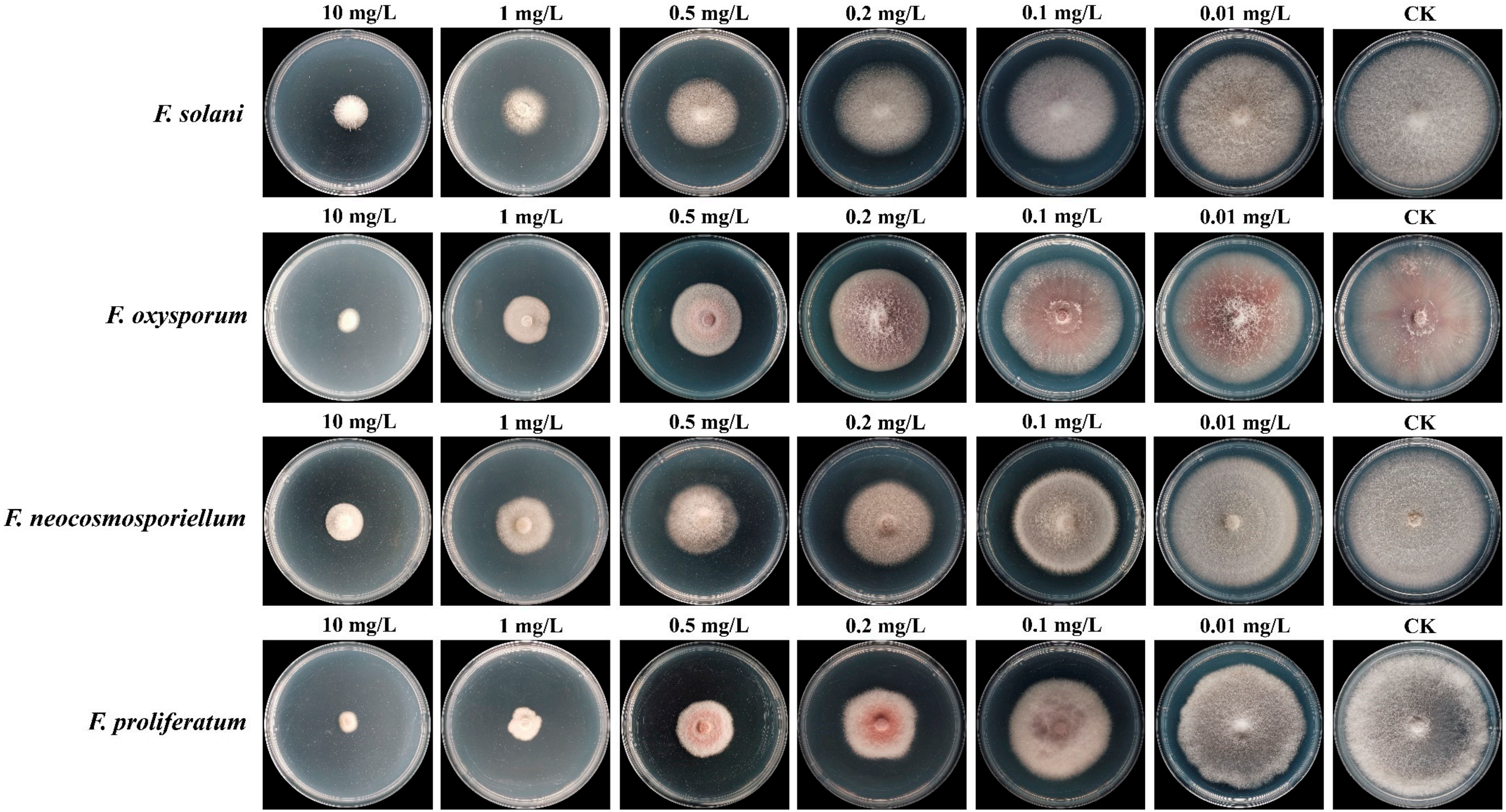

Figure S7. Effects of pydiflumetofen on the mycelial growth of the four *Fusarium* species (*F. solani*, *F. oxysporum*, *F. neocosmosporiellum,* and *F. proliferatum*).
